# Baicalin Attenuated A*β*_1-42_-Induced Apoptosis in SH-SY5Y Cells by Inhibiting the Ras-ERK Signaling Pathway

**DOI:** 10.1155/2022/9491755

**Published:** 2022-04-27

**Authors:** Zhenyan Song, Chunxiang He, Wenjing Yu, Miao Yang, Ze Li, Ping Li, Xu Zhu, Chen Xiao, Shaowu Cheng

**Affiliations:** ^1^Key Laboratory of Hunan Province for Integrated Traditional Chinese and Western Medicine on Prevention and Treatment of Cardio-Cerebral Diseases, Hunan University of Chinese Medicine, Changsha, Hunan 410208, China; ^2^College of Integrated Traditional Chinese and Western Medicine, Hunan University of Chinese Medicine, Changsha, Hunan 410208, China

## Abstract

Alzheimer's disease (AD) is a serious neurodegenerative disease. It is widely believed that the accumulation of amyloid beta (A*β*) in neurons around neurofibrillary plaques is the main pathological characteristic of AD; however, the molecular mechanism underlying these pathological changes is not clear. Baicalin is a flavonoid extracted from the dry root of *Scutellaria baicalensis Georgi*. Studies have shown that baicalin exerts excellent anti-inflammatory and neuroprotective effects. In this study, an AD cell model was established by exposing SH-SY5Y cells to A*β*_1-42_ and treating them with baicalin. Cell survival, cell cycle progression, and apoptosis were measured by MTT, flow cytometry, and immunofluorescence assays, respectively. The expression levels of Ras, ERK/ERK phosphorylation (p-ERK), and cyclin D1 were measured by Western blotting. In addition, whether the MEK activator could reverse the regulatory effect of baicalin on Ras-ERK signaling was investigated using Western blotting. We found that baicalin improved the survival, promoted the proliferation, and inhibited the apoptosis of SH-SY5Y cells after A*β*_1-42_ treatment. Baicalin also ameliorated A*β*_1-42_-induced cell cycle arrest at the S phase and induced apoptosis. Furthermore, baicalin inhibited the levels of Ras, p-ERK, and cyclin D1 induced by A*β*, and this effect could be reversed by the MEK activator. Therefore, we suggest that baicalin may regulate neuronal cell cycle progression and apoptosis in A*β*_1-42_-treated SH-SY5Y cells by inhibiting the Ras-ERK signaling pathway. This study suggested that baicalin might be a useful therapeutic agent for senile dementia, especially AD.

## 1. Introduction

Alzheimer's disease (AD) is a progressive, severe, and fatal neurodegenerative disease that is caused by aging. Cognitive dysfunction, especially memory impairment and impaired visual-spatial skills, is a major characteristic of AD. With the increasing size of the aging population, the prevalence of AD in the elderly population has increased over the years, and the prevalence rate of AD among 65-year-old Chinese people is as high as 6.6% [1]. At present, it is generally accepted that the major pathological characteristics of AD are extracellular neurofibrillary plaques and intracellular neurofibrillary tangles, but the molecular mechanism underlying the formation of these pathological characteristics is not clear [2]. Amyloid-beta (A*β*) deposition in the brain is thought to be the main pathological process underlying AD development [3]. The active subunit of *γ*-secretase cleaves amyloid precursor protein (APP) to produce A*β* [4]. A*β* and hyperphosphorylated tau coexist to cause a hyperactivity phenotype and lead to dysregulated expression of synaptic genes associated with synaptic function [5].

Primary neurons exposed to A*β*_1-42_ exhibited abnormal expression of cell cycle markers, DNA replication markers, and mitotic mutations [6]. Studies have shown that A*β* stimulation promotes the abnormal expression of factors related to cell cycle progression, such as inhibitor of differentiation-1 (Id1), sonic hedgehog (SHH), and cyclin D1, in neurons. Abnormal activation of the cell cycle in neurons involved in terminal differentiation leads to embrittlement degeneration and neuronal death [7]. A*β* can also induce increased reactive oxygen species (ROS) production and DNA damage in neurons, resulting in decreased expression of cyclin-dependent kinase 5 (Cdk5), which prevents cell cycle reentry in neurons [8, 9]. In response to costimulation with A*β*, overexpression of cyclin D1, which is a cell cycle promoter, activates the phosphorylation of retinoblastoma protein (Rb), and then, cells transition to the S phase. Excessive DNA damage leads to the activation of poly ADP-ribose polymerase (PARP) and p38 (Thr 180/Tyr 182), resulting in increased caspase-3 activation, increased Bax expression, and decreased Bcl-2/Bax ratios, leading to neuronal apoptosis [9]. This type of neuronal cell death that occurs between G1 and S before cells enter the synthetic phase of the cell cycle is classically called “abortive cell cycle reentry” and is characterized by upregulation of cell cycle- and apoptosis-related protein expression [10, 11]. Ras expression was enhanced, and ERK1/2 was activated in the cell model expressing APP [12]. A*β*_1-42_ can enhance the Ras-ERK signaling cascade. A*β*_1-42_ activates the Ras-ERK signaling pathway, which affects apoptosis and cell cycle progression and is involved in the occurrence of AD [13]. Therefore, the study of drugs that interfere with the signaling pathway of A*β* to prevent disorders in cell cycle progression and neurodegeneration in AD has certain clinical significance.

Baicalin, a flavonoid extracted from the dry root of *Scutellaria baicalensis Georgi*, has antiviral, antitumor, hypoglycemic, and lipid-lowering effects, along with brain-protecting, liver-protecting, and other effects, especially anti-inflammatory and neuroprotective effects [14–17]. Baicalin can reduce the microglial-mediated neuroinflammatory response and improve cognitive impairment in APP/PS1 mice [18], and baicalin alleviates Alzheimer's disease and memory deficits caused by the A*β*_1-42_ protein in rats [19]. In this study, human neuroblastoma cells (SH-SY5Y) exposed to A*β*_1-42_ were used to establish an AD cell model, and the effects of baicalin on cell cycle progression and apoptosis through the Ras-ERK signaling pathway were studied.

## 2. Methods

### 2.1. Cell Culture

Human neuroblastoma SH-SY5Y cells (CL-0208) were obtained from Procell (Wuhan, China) and grown in complete medium consisting of Eagle's MEM (11095080, Thermo Fisher, China) and Ham's F-12 Nutrient Mix (11765062, Thermo Fisher, China) supplemented with 10% FBS (10099, Thermo Fisher, China) and 100 U/mL penicillin–streptomycin (15070063, Thermo Fisher, China).

### 2.2. Experimental Design

A*β*_1-42_ (03112, Thermo Scientific, USA) was dissolved in saline (0.22 nmol/*μ*L) (in 0.1% DMSO). Aggregated A*β*_1-42_ was obtained by incubating the cells in a 37°C water bath for 7 days. SH-SY5Y cells in logarithmic growth were seeded in 96-well plates and incubated for 24 h. A*β*_1-42_ (03112, Thermo Scientific, USA, 10 *μ*M) was used to construct an AD cell model. Cell viability (MTT) and lactate dehydrogenase (LDH) activity (M8180 and BC0685, Solarbio, China) were measured at 6 h, 12 h, 24 h, and 36 h, and the optimal duration of treatment with A*β*_1-42_ was evaluated.

The cells were divided into a control group, a model group (10 *μ*M A*β*_1-42_), and baicalin low-, medium-, and high-dose treatment groups (5 *μ*M, 10 *μ*M, and 20 *μ*M).

### 2.3. Cell Viability and LDH Activity

SH-SY5Y cells were cultured and seeded in 96-well plates and grown to 80% (Lianke, China). The cells were collected, and 10 *μ*M A*β*_1-42_ for 24 h, the cell viability and LDH activity of each well were tested according to the instructions of the MTT kit and the LDH activity kit.

### 2.4. TUNEL Assay

SH-SY5Y cells were cultured and seeded in 6-well plates with coverslips at a concentration of 4 × 10^5^ cells/well. After 24 h of test processing, the cells were fixed with 4% paraformaldehyde for 10 min, washed with PBS, and permeabilized with 0.3% Triton X-100 for 5 min. TUNEL reagent (C1086, Beyotime, China) was added, the fixed cells were incubated at 37°C for 1 h, and the cells were then incubated with DAPI for 5 min to stain the nuclei, followed by a PBS wash. Confocal scanning microscopy (A1^+^, NIKON, Japan) was performed to image the cells with a 10× objective lens. Five fields were randomly selected, the average fluorescence intensity of each group was analyzed by ImageJ (Version 1.52 s), and the apoptosis rate was calculated.

### 2.5. Analysis of Cell Cycle Progression and Apoptosis by Flow Cytometry

SH-SY5Y cells were seeded in 6-well plates at a density of 4 × 10^5^ cells/well. After 24 h of experimental treatment, cell cycle progression was analyzed according to the method described in the Cell Cycle Staining Kit (CCS012, Lianke, China). The cells were collected, and 1 mL of room-temperature PBS was added. 3 mL of anhydrous ethanol (precooled at -20°C) was slowly added to the cells and simultaneously mixed at high speed. The cells were fixed overnight at -20°C. On the day of the test, 5 mL of room-temperature PBS was added. The cells were allowed to rehydrate for 15 minutes. One milliliter of PI dye was added to each tube and incubated in the dark for 30 min. The lowest loading speed was selected, and the cells were analyzed on a flow cytometer (MoFlo XDP, Beckman Coulter, Germany) at an excitation wavelength of 488 nm and detection channel of FL2-RPE (emission: 578 nm). The results were analyzed by Modfit LT (Version 5.0).

Following the instructions of the Annexin V-FITC Apoptosis Detection Kit (BMS500FI, Thermo Fisher, China), the cells were washed in PBS with gentle shaking. The cells were resuspended in 200 *μ*L of binding buffer (1×) at a cell density of 5 × 10^5^ cells/mL. Then, 5 *μ*L of Annexin V-FITC was added to 195 *μ*L of cell suspension and incubated for 10 min. The cells were washed in 200 *μ*L of binding buffer (1×) and resuspended in 190 *μ*L of binding buffer (1×). Finally, 10 *μ*L of propidium iodide (20 *μ*g/mL) was added, and FACS analysis was performed.

### 2.6. Western Blotting

Following the experimental treatment, the cells were lysed with a RIPA lysis buffer kit (Beyotime Biotechnology Co., Ltd., cat. P0013B, Shanghai, China) supplemented with 1% protease inhibitor and 1% phosphatase inhibitor. After sonicating at 30 Hz for 10 s to fragment the DNA, the samples were centrifuged at 12,000 rpm for 15 min to obtain the supernatants. A Pierce™ BCA Protein Assay Kit (cat. 23227, Thermo Scientific, Shanghai, China) was used to measure the protein concentrations. The samples were loaded into 8% SDS-PAGE gels and electrophoretically transferred to 0.45 *μ*m PVDF membranes (Millipore Sigma Inc., IPVH00010, Billerica, USA). Five percent nonfat dry milk or 5% BSA (V900933, Sigma-Aldrich, Billerica, USA) was added for blocking, and the primary antibodies (1: 1000) were incubated overnight at 4°C, followed by incubation with the secondary antibodies (1 : 10 000) at 37°C for 1 hour. The blots were treated with ECL Plus™ Western blotting substrate (32132, Thermo Scientific Technology, Shanghai, China) and then assessed using an imaging system (ChemiDoc™ XRS+, Bio-Rad, California, USA). The rabbit anti-Ras (ab52939), rabbit anti-ERK1+ERK2 (ab184699), and rabbit anti-phospho-ERK1+ERK2 (phospho T202+Y204) (ab214362) primary antibodies were purchased from Abcam (Cambridge, USA). The rabbit anti-cyclin D1 antibody (bs-0623R) and rabbit anti-*β*-actin (bs-0061R) primary antibodies were purchased from Bioss Biotechnology Co., Ltd. (Beijing, China). The goat anti-rabbit IgG antibody (cat. AP132P) was purchased from Sigma-Aldrich, Inc. (Billerica, USA).

### 2.7. Activator Treatment

A*β*_1-42_-treated SH-SY5Y cells were treated with the MEK activator PAFC-16 (20 *μ*M, Santa Cruz Biotechnology cat. sc-201009, Dallas, TX, USA), or 20 *μ*M baicalin for 24 h. Cell lysates were prepared and examined to assess changes in the ERK, P-ERK, and cyclin D1 levels by Western blotting analysis.

### 2.8. Statistical Analysis

Statistical Product and Service Solutions (SPSS, Version 21.0) was used for data statistics and analysis. The measurement data are expressed as the mean ± standard deviation (SD). A one-way ANOVA was conducted and normal analysis and homogeneity of variance tests. The LSD test was used to compare the square variances between groups; otherwise, Tamhane's T2 method was used. *P* values < 0.05 were considered significant.

## 3. Results

### 3.1. Baicalin Improved the Survival of A*β*_1-42_-Treated SH-SY5Y Cells

**Figure 1:**
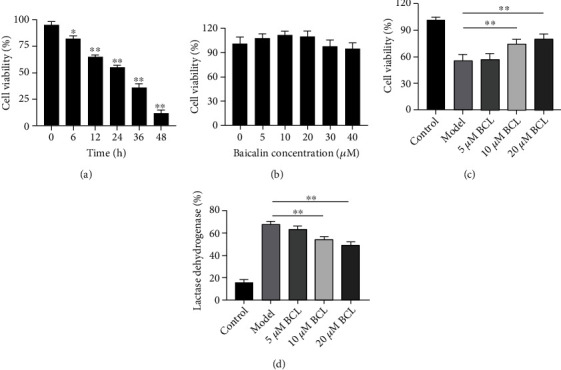
Baicalin improved the survival of A*β*_1-42_-treated SH-SY5Y cells. (a) Cell viability of SH-SY5Y cells treated with 10 *μ*M A*β*_1-42_ for different times. (b) Cell viability of SH-SY5Y cells treated with different concentrations of baicalin. (c) Cell viability of A*β*_1-42_-treated SH-SY5Y cells treated with different concentrations of baicalin. (d) LDH of A*β*_1-42_-treated SH-SY5Y cells treated with different concentrations of baicalin. The levels of cell viability and LDH are presented as the mean ± SD. *N* = 6, ∗ represents *P* < 0.05, and ∗∗ represents *P* < 0.01.

According to reports in the literature, we selected 10 *μ*M A*β*_1-42_ to induce SH-SY5Y cell injury and used MTT to observe the cell survival rate after A*β*_1-42_ treatment for different time points. The results showed that SH-SY5Y cell death occurred 6 hours after A*β*_1-42_ treatment, the cell survival rate decreased to 60% after 12 hours, and the cell survival rate at 24 hours was close to 50% (Figure 1(a)). Therefore, we chose to treat SH-SY5Y cells with A*β*_1-42_ for 24 hours to establish an AD cell model.

In a preliminary study, we assessed the cytotoxicity of baicalin. We found that the concentrations of baicalin tested (5 *μ*M, 10 *μ*M, 20 *μ*M, 30 *μ*M, and 40 *μ*M) did not cause SH-SY5Y cell death (Figure 1(b)). In the following experiment, we evaluated the pharmacodynamic effect of baicalin. Cell viability was measured by the MTT method, and cell membrane damage was assessed by measuring LDH leakage (LDH %). The MTT test results (Figure 1(c)) showed that compared with the model group, treatment with 5 *μ*M baicalin improved cell viability to a certain extent after 24 h (*P* < 0.05), and treatment with 10 *μ*M and 20 *μ*M baicalin significantly improved the viability of A*β*_1-42_-treated SH-SY5Y cells after 24 h (*P* < 0.05). The LDH leakage rate results (Figure 1(d)) showed that compared with the model group, baicalin reduced the LDH leakage rate of cells in the model group to a certain extent, and the difference between cells treated with 10 *μ*M and 20 *μ*M baicalin for 24 h was considered to be statistically significant (*P* < 0.05).

### 3.2. Baicalin Inhibited A*β*_1-42_-Induced SH-SY5Y Cell Apoptosis

**Figure 2:**
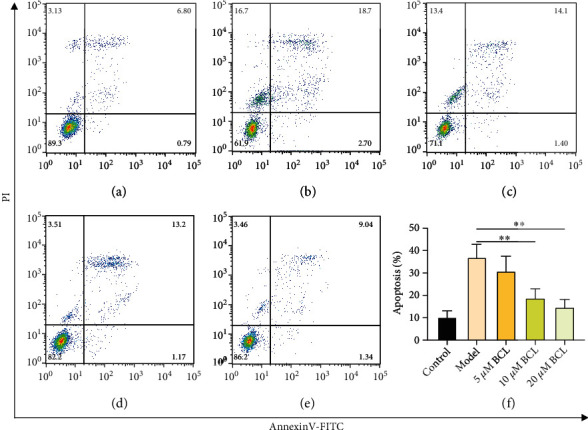
Effect of baicalin on A*β*_1-42_-treated SH-SY5Y cell apoptosis. (a) Control group. (b) Model group (10 *μ*M A*β*_1-42_). (c) 5 *μ*M baicalin treatment group. (d) 10 *μ*M baicalin treatment group. (e) 20 *μ*M baicalin treatment group. (f) Apoptosis rate. The apoptosis rate is presented as the mean ± SD. *N* = 5; ∗∗ represents *P* < 0.01.

Phosphatidylserine (PS) is usually located inside the cytoplasmic membrane facing the cytoplasm. However, when apoptosis occurs, the asymmetry of the cell membrane is disrupted, and PS is localized to the cell surface [20]. This biochemical event is known as a marker of apoptosis. In this study, we used Annexin V-FITC/propidium iodide (PI) staining and flow cytometry to investigate the externalization of PS induced by A*β*_1-42_. The apoptosis results showed that baicalin inhibited A*β*_1-42_-mediated SH-SY5Y cell apoptosis in a concentration-dependent manner (Figures 2(a)–2(e)). The statistical analysis results of fluorescence intensity showed that, compared with the control group, the apoptosis rate of SH-SY5Y cells treated with 10 *μ*M A*β*_1-42_ for 24 h was increased significantly (*P* < 0.05), and the apoptosis rate was 38.12 ± 6.28%. Compared with the model group, the incidence rates of apoptosis after treatment with 10 *μ*M and 20 *μ*M baicalin were 18.69 ± 4.26% and 14.64 ± 4.15%, respectively, and the differences between these values were statistically significant (*P* < 0.05).

**Figure 3:**
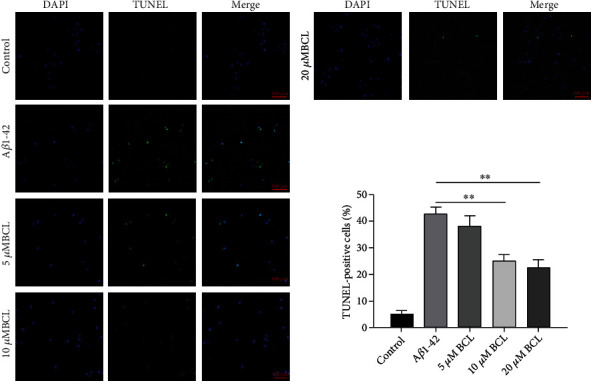
A*β*_1-42_-induced apoptosis in SH-SY5Y cells was inhibited by baicalin treatment. SH-SY5Y cells were treated with 5 *μ*M, 10 *μ*M, and 20 *μ*M baicalin for 24 h and analyzed by TUNEL fluorescence staining with a NIKON A1^+^ confocal microscope. The experiment was repeated twice. A total of 100 DAPI-positive nuclei were counted from three separate fields, and the percentage of apoptosis was calculated based on the number of TUNEL-positive nuclei in each field. The TUNEL-positive rate is presented as the mean ± SD. *N* = 5; ∗ represents *P* < 0.05.

To determine whether baicalin treatment interferes with A*β*_1-42_-induced apoptosis in SH-SY5Y cells, a TUNEL apoptosis detection kit was used to analyze SH-SY5Y cell apoptosis. SH-SY5Y cells were treated with or without 10 *μ*M A*β*_1-42_ and were treated with or without baicalin for 24 h. Analysis of SH-SY5Y cells showed that these drugs significantly inhibited A*β*_1-42_-induced SH-SY5Y apoptosis (Figure 3). These data suggest that baicalin protects SH-SY5Y cells against A*β*_1-42_-mediated cytotoxicity.

### 3.3. Baicalin Ameliorated A*β*_1-42_-Induced SH-SY5Y Cell Cycle Arrest in the S Phase

**Figure 4:**
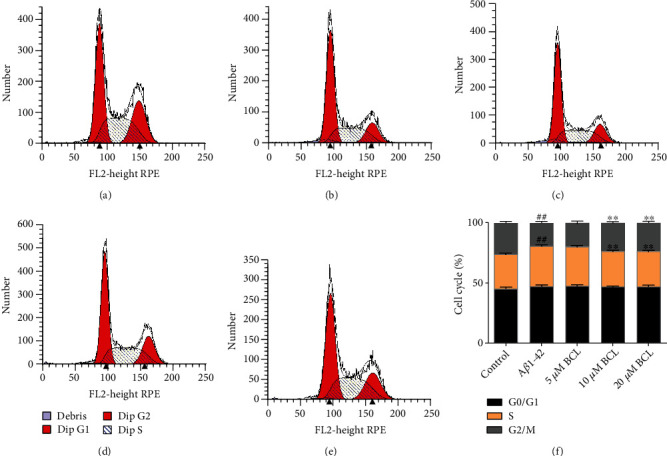
Effect of baicalin on the cell cycle of A*β*_1-42_-treated SH-SY5Y cells. (a) Control group. (b) Model group (10 *μ*M A*β*_1-42_). (c) 5 *μ*M baicalin treatment group. (d) 10 *μ*M baicalin treatment group. (e) 20 *μ*M baicalin treatment group. (f) The data are presented as the mean ± SD. *N* = 5; compared with the model group, ## represents *P* < 0.01; compared with the model group, ∗∗ represents *P* < 0.01.

A*β* plays a significant role in different cellular processes, such as proliferation, apoptosis, and signal transduction [21]. Then, the effect of baicalin on SH-SY5Y cell cycle progression after treatment with A*β*_1-42_ was measured by flow cytometry (Figures 4(a)–4(e)). Compared with the control, 24 h of treatment with 10 *μ*M A*β*_1-42_ significantly increased the number of SH-SY5Y cells in the model group in the S phase (*P* < 0.05) and significantly decreased the number of cells in the G2/M phase (*P* < 0.05). Compared with the model group, the 10 *μ*M and 20 *μ*M baicalin treatment groups had an obvious decrease in the number of cells in the S phase (*P* < 0.05), with a significant increase in the number of cells in the G2/M phase (*P* < 0.05). In the 5 *μ*M baicalin intervention group, there were no significant differences in the numbers of cells in the G0/G1, S, and G2/M phases of the cell cycle. The results showed that a large number of SH-SY5Y cells were arrested in the S phase after treatment with A*β*_1-42_. The effect of A*β*_1-42_ on SH-SY5Y cell cycle progression was ameliorated by 10 *μ*M and 20 *μ*M baicalin treatment.

### 3.4. Baicalin Inhibited the A*β*_1-42_-Induced Activation of Ras-ERK Signaling

**Figure 5:**
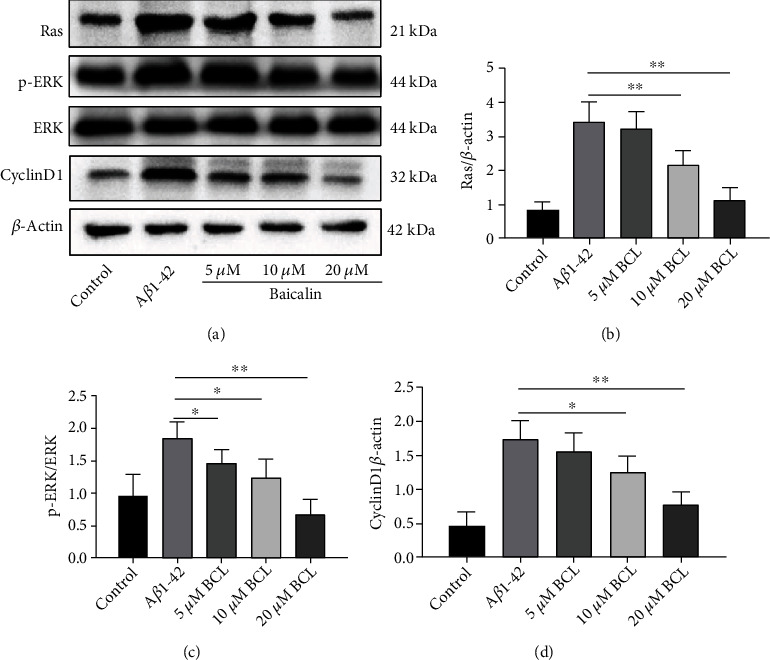
Inhibition of Ras-ERK signaling pathway activation in A*β*_1-42_-treated SH-SY5Y cells by baicalin. (a) SH-SY5Y cell extracts were prepared and analyzed by Western blotting using Ras, P-ERK, total ERK, and cyclin D1 antibodies. *β*-Actin antibodies were used as a reference. (b) Ras protein expression normalized to *β*-actin. (c) p-ERK levels normalized to total ERK expression. (d) Cyclin D1 expression normalized to *β*-actin. The protein expression is presented as the mean ± SD. *N* = 3, ∗ represents *P* < 0.05, and ∗∗ represents *P* < 0.01.

The Ras-ERK pathway can stimulate cyclin D1 expression and regulate cell cycle progression. Finally, we studied whether the above regulation has an impact on the Ras-ERK pathway; thus, we evaluated the effect of A*β*_1-42_ and baicalin on the p-ERK1/2 and cyclin D1 protein levels. After 24 h of baicalin treatment, the cells were harvested, and the protein expression levels were detected by Western blotting. The Western blotting results (Figures 5(a)–5(d)) showed that compared with the blank control group, the SH-SY5Y cells in the A*β*_1-42_ treatment group exhibited significantly increased Ras protein expression levels, and the p-ERK1/2 and cyclin D1 protein levels were significantly upregulated (*P* < 0.05). Compared with the model group, the protein expression levels of Ras, p-ERK1/2, and cyclin D1 decreased after treatment with 5 *μ*M baicalin, but the difference was not statistically significant. The protein expression levels of Ras, p-ERK1/2, and cyclin D1 were significantly decreased after treatment with 10 *μ*M and 20 *μ*M baicalin (*P* < 0.05).

### 3.5. The Effect of Baicalin on the Ras-ERK Signaling Axis by the MEK Activator

**Figure 6:**
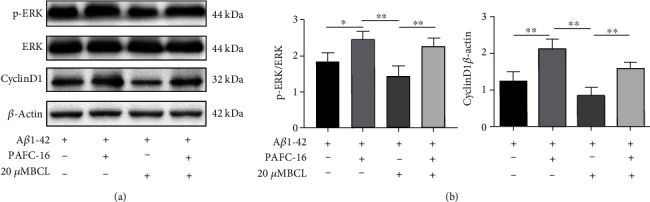
Baicalin inhibited Ras-ERK signaling and cyclin D1 expression in A*β*_1-42_-treated SH-SY5Y cells, and this effect was reversed with a MEK activator. (a) SH-SY5Y cell extracts were prepared and analyzed by Western blotting using P-ERK, total ERK, and cyclin D1 antibodies. *β*-Actin antibodies were used as a reference. (b) p-ERK levels normalized to total ERK expression; cyclin D1 expression normalized to *β*-actin. The protein expression is presented as the mean ± SD. *N* = 3, ∗ represents *P* < 0.05, and ∗∗ represents *P* < 0.01.

We determined whether baicalin ameliorated A*β*_1-42_-induced SH-SY5Y cell damage, which may be related to the inhibition of the Ras-ERK signaling pathway. We conducted studies on A*β*_1-42_- treated SH-SY5Y cells treated with or without the ERK activator PAFC-16. Our results showed that PAFC-16 further increased the phosphorylated ERK levels in A*β*_1-42_-treated SH-SY5Y cells. A*β*_1-42_-treated SH-SY5Y cells treated with PAFC-16 showed an increase in the levels of cyclin D1, demonstrating that this activation occurs downstream of Ras-ERK activation. Furthermore, FAFC-16 counteracted the baicalin-induced inhibition of ERK phosphorylation and cyclin D1 expression (Figures 6(a)–6(b)). The results suggested that baicalin ameliorated A*β*_1-42_-induced SH-SY5Y cell damage by inhibiting the Ras-ERK axis.

## 4. Discussion

A*β* aggregation and plaque formation are important factors in the pathogenesis of AD. A*β* toxicity induces abnormal activation of the cell cycle in terminally differentiated neurons, which is an important cause of neuronal apoptosis [7]. The use of SH-SY5Y cells to study the damage caused by toxic stimulation with A*β* on nerve cells is the most commonly used cell model to evaluate neuronal apoptosis in AD. Several studies have shown that the decrease in cell viability and toxic damage occur in dose-dependent manners in A*β*-induced SH-SY5Y cell models, including oxidative stress injury, mitochondrial dysfunction, decreased bcl-2/Bax expression, increased cytochrome C release, and increased caspase-3 activity [22–24]. In addition, studies have shown that SH-SY5Y exposed to A*β*-induced toxicity can alter cell cycle progression by activating MAPK-ERK1/2, significantly increasing the number of cells in the S phase and decreasing the number of cells in the G2/M region [25, 26]. In this study, A*β*-induced toxicity promoted the reentry of SH-SY5Y cells into the cell cycle, arrested the cells in the S phase, and induced apoptosis; these effects were related to the activation of the Ras-ERK signaling pathway. Baicalin treatment can reverse this phenomenon to a certain extent.

In recent years, the potential neuroprotective effects of the active components of herbal extracts on various neurological diseases have been extensively studied [27, 28]. Baicalin has a variety of biological properties, including anti-inflammatory, antioxidant, and anticancer activities. Numerous preclinical trials have demonstrated baicalin's potential for treating a wide range of human diseases [29–31]. Increasing evidence suggests that baicalin can be used as a potential anti-AD drug, playing a key role in inhibiting A*β*-induced neurotoxicity. Yu et al. [32] found that baicalin inhibited the formation of A*β*_1-42_ fibers and increased the viability of cells after incubation with A*β*_1-42_ by UHPLC-DAD-TOF/MS. Baicalin can effectively improve the learning and memory impairment, hippocampal injury, and neuronal apoptosis induced by A*β*_1-42_ injection in rats, and this effect may be related to the antioxidant effect mediated by Nrf2 [33]. Another study showed that baicalin reduced memory and cognitive deficits in APP/PS1 mice by inhibiting activation of the NLRP3 inflammasome and the TLR4/NF-*κ*B signaling pathway to inhibit microglia-induced neuroinflammation [18]. In vitro experiments also proved that baicalin exerts a good protective effect on A*β*-induced cell damage. Baicalin directly interacts with copper to inhibit the accumulation of A*β*_1-42_ and protects SH-SY5Y cells from the oxidative damage caused by A*β*_1-42_ accumulation by reducing the production of H_2_O_2_ [34]. Baicalin-mediated activation of Nrf2 showed a strong ability to resist oxidative stress in N2a/APPswe cells [35]. In addition, baicalin can inhibit A*β*-induced microglial activation [36] and reduce A*β* cytotoxicity in PC12 cells [37]. Our results showed that baicalin can inhibit A*β* to cause cell cycle disorders and apoptosis, which may be related to the overactivation of the Ras-ERK signaling pathway.

G1/S-specific cyclin D1 (cyclin D1) is a protein encoded by the human CCND1 gene. The cyclin D1 gene is a part of the highly conserved cell cycle family. Cyclin D1 regulates cyclin-dependent kinases (CDKs). Different cyclins exhibit unique expression and degradation characteristics, which contribute to the temporal coordination of cell cycle events [38]. Cyclin D1 is associated with cell cycle activation and G1/S progression. A*β* promotes the expression of cyclin D1 and enables cells to escape anaphase in mitosis and reenter the cell cycle [25]. However, adult neurons no longer enter the cell cycle (remaining in the G0 phase) and are considered permanent postmitotic cells. In the brains of AD patients, the accumulation of cyclin D1 was associated with cell cycle activation, which eventually leads to cell death [39]. Increased Ras expression is associated with the cell cycle and enhances the Ras-ERK signaling axis to activate AP-1 and induce cyclin D1 expression [40]. However, the overactivation of the Ras-ERK pathway can block the cell cycle and result in excessive accumulation of cyclin D1 in cells, which can prevent the degradation of the cell cycle inhibitor p21CIP1 and cause the cells to enter a static state [41]. A*β* can not only activate Ras-ERK signaling but also enhance the expression and nuclear accumulation of cyclin D1 in neurons. Inhibition of Ras-ERK can induce cyclin D1 expression and nuclear accumulation to weaken the cascade of ERK signals [13]. The results of cell cycle analysis by flow cytometry showed that the numbers of SH-SY5Y cells in the S phase were significantly increased and the numbers of cells in the G2/M phase were significantly decreased after treatment with A*β*_1-42_, indicating cell cycle arrest. After baicalin treatment, the numbers of cells in the S phase were significantly reduced. Moreover, the WB results showed that Ras-ERK signaling pathway activation was inhibited. These results suggest that baicalin treatment could improve the effect of A*β*_1-42_ on SH-SY5Y cell cycle progression. We hypothesized that the possible mechanism was that the stimulation of neurons with A*β* forced the neurons into the cell cycle. However, cell cycle dysfunction successfully prevented neuronal division or made the cells vulnerable, which led to neuronal degeneration and eventually neuronal death, while baicalin could effectively alleviate the cell cycle arrest caused by A*β*.

Deposition of A*β* is considered to be the central link in the pathogenesis of AD, and the pathological process of A*β* is closely related to the Ras-ERK signaling pathway [13]. The Ras-ERK signaling pathway can transduce extracellular signals to the nucleus and affect cell fate, including cell proliferation, differentiation, survival, and transformation [42]. Activation of this pathway under different conditions might trigger cell-specific responses. In general, sustained and intense activation of this pathway increases cell proliferation by promoting protein synthesis and the formation of cyclin/cyclin-dependent kinase (CDK) complexes [43]. Abnormal activation of the Ras-ERK pathway can induce cell apoptosis and is of great significance to the pathogenesis of AD and other degenerative diseases [44]. Many types of cells are characterized by dysfunction of cell cycle regulation in AD, including neurons. Abnormal release of growth factors, cytokines, and other molecules, such as nitric oxide, oxygen free radicals, or lipid peroxidation products in the mitotic signaling pathway, such as the MAPK signaling pathway, leads to excessive stimulation of mitotic neurons, resulting in abnormal cell division and dedifferentiation and neuronal death in terminally differentiated neurons [45]. In AD, abnormal cell cycle activation and neuronal loss induced by A*β* oligomers may be associated with Ras [46]. Studies have shown that statins can improve cognitive function in mice with AD by inhibiting the activity of Ras [47]. Our results showed that Ras expression at the cellular level was increased after treatment with A*β*_1-42_. The significant upregulation of p-ERK expression indicated that it was activated. Moreover, the expression level of SH-SY5Y cyclin D1 was also increased after treatment with A*β*_1-42_, suggesting that A*β* activation of the Ras-ERK signaling pathway may be one of the mechanisms by which A*β* induces cell cycle disorders and apoptosis, leading to neurodegeneration and neuronal loss in AD. This is in accordance with the previous literature [48]. However, after treatment with 10 *μ*M and 20 *μ*M baicalin, the Ras, p-ERK, and cyclin D1 levels in SH-SY5Y cells exposed to A*β*_1-42_ were significantly decreased, suggesting that the inhibition of the proapoptotic ERK pathway was involved in the neuroprotective effect of baicalin. Additionally, we hypothesized that baicalin may also exert therapeutic effects through other pathways.

## 5. Conclusion

In this study, an AD cell model was established by treating SH-SY5Y cells with A*β*_1-42_, and baicalin was administered for intervention. The results suggested that baicalin can significantly reduce the SH-SY5Y cell damage induced by A*β*_1-42_, change the cell cycle composition ratio and inhibit cell apoptosis, which may be related to the inhibition of the Ras-ERK signaling pathway.

## Data Availability

The datasets used and/or analyzed during the current study are available from the corresponding author on reasonable request.
